# Chemical and Morphological Constitutive Defensive Traits of Cyanobacteria Have Different Effects on the Grazing of a Small Tropical Cladoceran

**DOI:** 10.3390/toxins17070343

**Published:** 2025-07-05

**Authors:** Luciana Machado Rangel, Marcella Coelho Berjante Mesquita, Shara Rosa de Barros, Vinicius Neres-Lima, Michael Ribas Celano, Mauro Cesar Palmeira Vilar, Sandra Maria Feliciano de Oliveira e Azevedo, Marcelo Manzi Marinho

**Affiliations:** 1 Laboratory of Ecology and Physiology of Phytoplankton, Department of Plant Biology, University of Rio de Janeiro State, Rua São Francisco Xavier 524—PHLC Sala 511a, Rio de Janeiro 20550-900, Brazil; shararoosa@outlook.com (S.R.d.B.);; 2 Laboratory of Ecology of River and Stream, Department of Ecology, University of Rio de Janeiro State, Rua São Francisco Xavier 524—PHLC Sala 220, Rio de Janeiro 20550-900, Brazil; vinicius.lima.eco@gmail.com; 3 Laboratory of Ecophysiology and Toxicology of Cyanobacteria, Carlos Chagas Filho Institute of Biophysics, Federal University of Rio de Janeiro, Rio de Janeiro 21949-902, Brazil; michaelribas@biof.ufrj.br (M.R.C.); maurovilar@biof.ufrj.br (M.C.P.V.); sazevedo@biof.ufrj.br (S.M.F.d.O.e.A.)

**Keywords:** cyanotoxin, harmful algal bloom, *Microcystis*, *Moina*, predator defense, *Planktothrix*, zooplankton

## Abstract

Antipredator defenses of bloom-forming cyanobacteria species maximize their fitness but can reduce carbon and energy transfer efficiency to higher trophic levels, making them a key regulator of plankton communities in eutrophic waters. We investigated the grazing responses of the tropical cladoceran *Moina micrura* to different strains of the cyanobacteria *Microcystis aeruginosa* and *Planktothrix isothrix*, using a good food source (green algae *Mono*-*raphidium capricornutum*) as a control. Both *Microcystis* strains grow as unicellular and are microcystins producers; however, this cyanotoxin was not detected on the filamentous *Planktothrix* strains. *M. micrura* ingested all cyanobacteria at reduced rates compared to single diets with *Monoraphidium*. In mixed diets, food type had a significant effect on grazing responses, which differed interspecifically. *Planktothrix* was more grazed than *Microcystis* strains. Feeding selectivity on *Monoraphidium* was negatively affected by the increase of cyanobacteria in the diet. We observed varied responses across treatments, ranging from feeding inhibition to different degrees of tolerance toward cyanobacteria, particularly in non-microcystin-producing species. We also highlight the selectivity of small tropical cladocerans, a pattern that is not yet well documented. These findings emphasize that studies incorporating phyto- and zooplankton with a history of coexistence can provide more meaningful insights into natural ecosystem dynamics.

## 1. Introduction

In recent decades, cyanobacterial blooms have become more frequent, lasting longer, and exhibiting more significant impacts [1]. They can be highly toxic, generate hypoxic conditions, and severely disrupt aquatic food chains, posing a serious threat to the integrity of freshwater resources [2]. Many bloom-forming species can produce a diverse range of toxic metabolites and bioactive compounds with harmful potential for animal and human health [3]. The hepatotoxic cyclic peptides microcystins are frequently found in fresh and brackish waters and are among the most reported cyanotoxins globally [4,5]. The planktonic genera *Microcystis* and *Planktothrix* are associated with the production of this group of cyanotoxin [6,7]. Microcystin-producing strains have been recorded in a wide diversity of habitats, including tropical, temperate, and polar environments, as well as extreme habitats [7].

Grazing by cladocerans, copepods, and rotifers could be an important factor in controlling blooms; however, cyanobacteria traits such as the ability to produce toxic metabolites, low nutritional quality, and morphology have been shown to inhibit such control [8,9,10]. Thus, in natural environments, the proliferation of cyanobacteria can affect the structure and composition of the zooplankton community, selecting smaller and/or more tolerant herbivores [11,12]. Due to the significance of aquatic energy flow, many ecologists have attempted to estimate the grazing process of zooplankton species [13]. Since grazing is one of the main processes of phytoplankton loss, understanding predation of zooplankton that co-occur with toxic cyanobacteria in nature can explain the contribution of this process for the occurrence and duration of blooms [8,14,15].

Phytoplankton has evolved a diverse array of defenses against zooplankton grazing, a critical selective pressure shaping their community structure [16,17]. These defenses can be constitutive or inducible [18]. Constitutive defenses are continuously expressed traits, regardless of the presence of grazers, offering a baseline level of protection. In contrast, inducible defenses are only activated or intensified in response to cues indicating grazer presence or activity, representing a more energy-efficient strategy and include alterations in morphology, the production of toxins, and changes in palatability. These defensive mechanisms are pivotal in mediating trophic interactions and ultimately influencing the success and bloom dynamics of various phytoplankton species, particularly in the context of harmful algal blooms [18,19].

Considering the dominance of smaller zooplankton in tropical freshwater, passive size-based food selection related to smaller predator body size can occur [15]. Phytoplankton that are too large to be consumed by small zooplankton become more prevalent. Thus, zooplankton can directly influence the phytoplankton community, by foraging on a specific size. For instance, many bloom-forming cyanobacteria are large filamentous or colonial species [17,20]. Those phytoplankton cells within the prey size spectra can experience greater predation pressure, while the ones that are over this range may increase in number [21,22]. As for bloom-forming cyanobacteria, large size and/or colonies with mucilage sheaths can impair ingestion and digestion by potential grazers [23,24]. Thus, prey size selection by grazers might facilitate the dominance of bloom-forming cyanobacteria [21].

Moreover, feeding selectivity based on nutritional quality and/or the presence of harmful metabolites is another mechanism that allows zooplankton to survive during harmful cyanobacterial blooms [9,10]. This selective behavior allows zooplankton to reduce their ingestion of potentially toxic prey and, in addition, might facilitate blooms by reducing the density of competing phytoplankton [25,26]. Furthermore, in environments where cyanobacterial blooms are frequent, some zooplanktonic organisms develop tolerance to these toxic cyanobacteria over several generations [27,28]. This local adaptation allows them to ingest the cyanobacteria and neutralize their toxic effects, ensuring their survival in ecosystems impacted by harmful blooms [29,30].

Although more information is available on the interactions between *Microcystis* and *Daphnia*, less is known about the interaction of cyanobacteria with small-bodied tropical zooplankton [21,31]. In some environments, toxic cyanobacterial blooms shift the size distribution of zooplankton communities, causing large-bodied species to be replaced by smaller ones like rotifers, and small cladocerans [12]. However, even smaller cladocerans, such as *Ceriodaphnia*, *Macrothrix* and *Moina* can present different responses to the same cyanobacteria [31,32]. The feeding pattern of most cladocerans is generally considered to be predominantly filter feeding, with passive suspension feeding behavior, which implies less selectivity in relation to prey [33,34,35,36]. However, positive selectivity coefficients in experimental studies were observed for some cladocerans, especially small ones, demonstrating some degree of selectivity for a few species [37,38]

For instance, the neotropical *Moina micrura* has shown different sensitivity responses to cyanobacteria in laboratory assays [32,38,39,40]. Previous studies have shown that this species could feed efficiently on a wide range of sizes and types of phytoplankton [38], as well as small filaments of cyanobacteria, but not colonies of *Microcystis aeruginosa* [40]. *Moina micrura* even showed increased fitness in diets with the filamentous non-saxitoxin producing *Raphidiopsis* [39]. This cladoceran also consumed single cells of *Microcystis aeruginosa* in significant amounts when it was provided as their sole food source [41]. Despite the importance of small cladocerans such as *Moina micrura* in high trophic levels, their grazing impact in bloom-forming cyanobacteria is less understood, especially in tropical regions [21,31]. More information about this species is essential for a more comprehensive understanding of zooplankton ecology in tropical ecosystems with a history of cyanobacteria blooms.

The bloom-forming genera *Microcystis* and *Planktothrix* are globally widespread and present notable morphological differences; *Microcystis* species are formed by numerous coccoid cells, which in nature are usually found grouped in large colonies surrounded by mucilage sheaths, although the occurrence of single cells has also been recorded [42,43]. On the other hand, the genus *Planktothrix* is represented by filamentous species that are straight or irregularly curved [44,45]. However, these two genera have adaptations in common, such as the presence of aerotopes, large size, and the ability to produce microcystins and other bioactive peptides, as well as deterrent volatile organic compounds [6,7,46,47].

*Microcystis aeruginosa* and *Planktothrix isothrix* are the dominant species of a shallow tropical eutrophic coastal lagoon (Jacarepaguá Lagoon, Brazil), where long-lasting cyanobacterial blooms are frequently observed. Together, they can represent more than 90% of the total phytoplankton biomass [48,49]. *Moina micrura* is among the dominant zooplankton that co-occur with these cyanobacteria blooms in Jacarepaguá Lagoon [49,50]. Therefore, this study aims to determine whether *Microcystis aeruginosa* and *Planktothrix isothrix* exhibit inter- and intraspecific differences in losses caused by grazing from *Moina micrura*. The cyanobacterial strains and cladoceran clone were isolated from the same environment (Jacarepaguá Lagoon, Brazil) and thus have a history of co-existence. In addition, the species used exhibit distinct functional traits with the potential to affect their grazing losses [22,51]. *Planktothrix isothrix* strains have variations in filament size, while the cultured *Microcystis aeruginosa* species have grown unicellular and vary in microcystin production. Therefore, we hypothesized that *Moina micrura* would exhibit reduced grazing rates on cyanobacteria compared to the edible green alga *Monoraphidium capricornutum*, and that this effect would intensify with increasing cyanobacterial density. Among cyanobacteria, we expected that strains producing microcystins and/or exhibiting larger filament size (e.g., *Planktothrix isothrix*) would result in greater feeding inhibition. In mixed diets, we expected that higher proportions of cyanobacteria would decrease total clearance rates and promote greater selectivity for *Monoraphidium capricornutum*, reflecting an avoidance of lower-quality food.

## 2. Results

### 2.1. Grazing on Different Amounts of Food (Single Diets)

**Figure 1:**
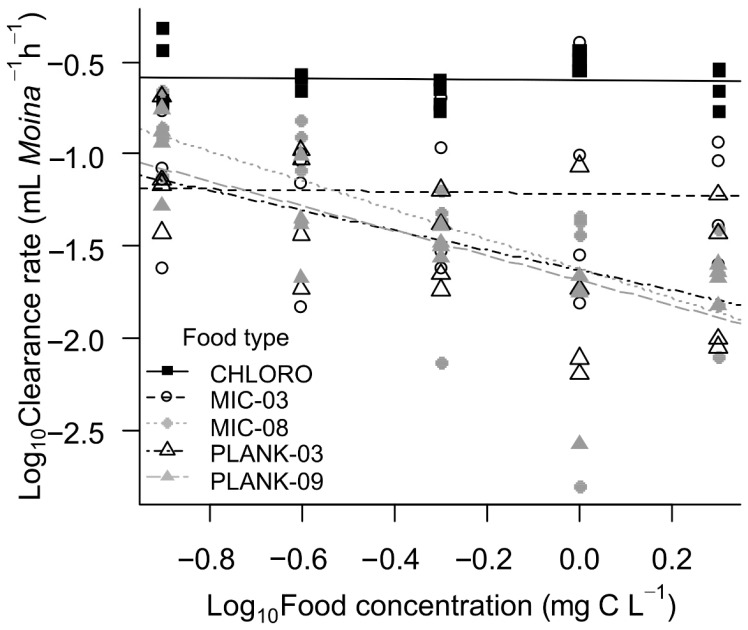
Clearance rates of *Moina micrura* (logarithmized data) on different concentrations (also logarithmized data) of *Monoraphidium* (Mono), *Microcystis aeruginosa* strains MIC 03 and MIC 08 and *Planktothrix isothrix* strains PLANK 03 and PLANK 09.

The overall mean clearance rate was 0.10 mL ± 0.10 *Moina*^−1^ h^−1^ with the lowest value in the treatment with the PLANK-09 diet at a concentration of 1 mg C L^−1^ (mean = 0.015 and sd = 0.008 mL *Moina*^−1^ h^−1^) and the highest value with the *Monoraphidium* diet at a concentration of 1 mg C L^−1^ (mean = 0.315 ± 0.040 mL *Moina*^−1^ h^−1^). Significant differences in the interaction between food type and concentration were found affecting the feeding behavior of *Moina* in terms of the clearance rates (GLM: F_4,90_ = 4.4; *p* < 0.01). Regarding the food type, the clearance rates (CRs) of *Monoraphidium* were significantly higher compared to the diets composed of cyanobacteria (Figure 1; Table 1). Independent of food concentration, the medians of CR values in *Monoraphidium* diets were always equal to or greater than 0.2 mL *Moina*^−1^ h^−1^, while in diets with cyanobacteria they were close to or below 0.1 mL *Moina*^−1^ h^−1^. However, there was no significant difference in the CRs of cladocerans between diets of microcystin-producing strains (*Microcystis)* and diet of large cyanobacteria (*Planktothrix*), except for the MIC-03 strain at higher concentrations (>1 mg C L^−1^). Furthermore, *Monoraphidium* concentration had no significant effect on CRs of cladocerans (Figure 1; Table 1).

**Figure 2:**
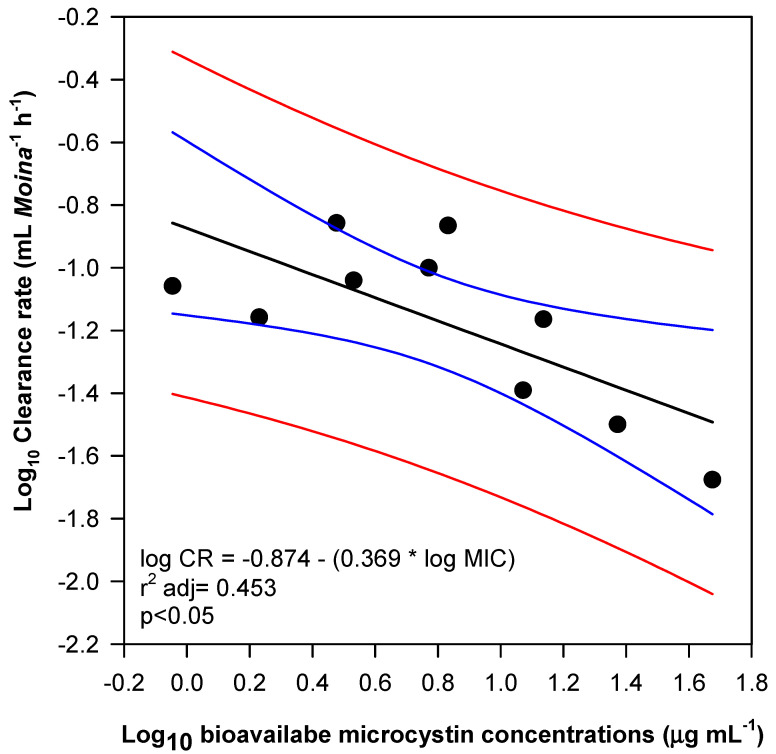
Linear regression of clearance rates against bioavailable microcystin (the product of the cellular quota and cell density for each carbon concentration). This analysis was performed with the averages of the CRs in Single Diets and data only from the microcystin-producing strains, MIC-03 and MIC-08. Red lines indicate sample confidence interval, blue lines the regression confidence interval and the black line the regression line.

Considering the potential impact of toxin availability in diets enriched with the microcystin-producing strains *Microcystis aeruginosa* MIC-03 and MIC 08, a linear regression analysis was performed on the cladoceran filtration rate and total bioavailable microcystin (as the total microcystin content in the different biomasses of *Microcystis* provided as food) in the single diets, which revealed a significant negative relationship between these variables (adjusted r^2^ = 0.453; *p* < 0.05), so that the increased microcystin level decreased *Moina* filtration rates (Figure 2).

### 2.2. Grazing on on Mixed Diets

**Figure 3:**
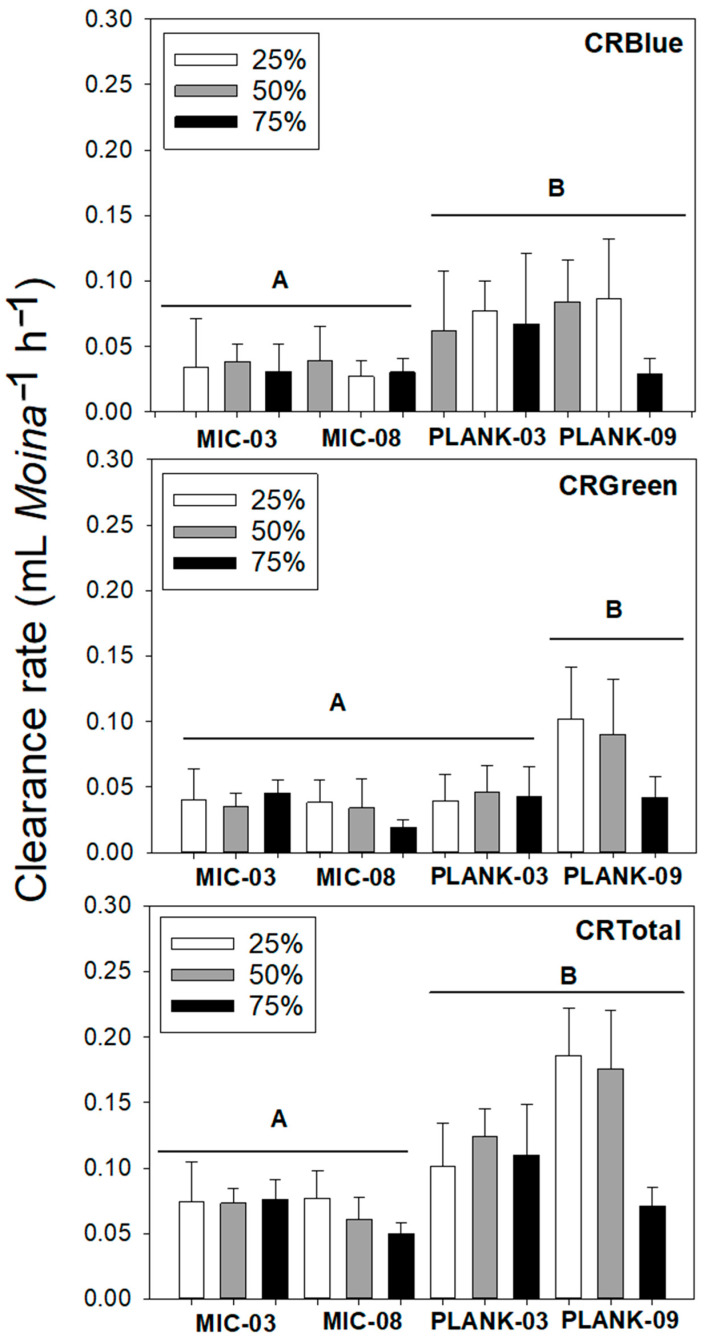
Clearance rates of *Moina micrura* on different proportions (75, 50 and 25%) of cyanobacteria (*Microcystis aeruginosa* strains MIC-03 and MIC-08 and *Planktothrix isothrix* strains PLANK-03. and PLANK-09) in combination with good food, *Monoraphidium*. Error bars indicate standard deviation (n = 4). Different letters mean significant differences, according to the tests described in Table 2.

In general, animals ingested less in the mixed suspension diets compared to the single diet trials, especially compared to the *Monoraphidium* diets. CRs on cyanobacteria only (CR_Blue_) in the mixed diets did not exceed 0.2 mL *Moina*^−1^ h^−1^ (Figure 3). For these grazing responses, initially, we performed a multiplicative model, considering the dominance of cyanobacteria, food type and their interaction, but the interaction was not significant (two-way ANOVA: F_6,36_ = 1.1, *p* = 0.401). Therefore, we opted for an additive model, removing the interaction factor. In this model, only food type was significant (two-way ANOVA: F_3,42_ = 4.8; *p* = 0.006). The *post hoc* Tukey HSD tests highlighted differences between *Microcystis* and *Planktothrix*, but not between the strains of each of these species (Figure 3, Table 2).

As for CR_Blue_, the CRs on *Monoraphidium* in different proportions with cyanobacteria (CR_Green_) we also elected an additive model, removing the interaction factor as this was not significant (two-way ANOVA: F_6,36_ = 0.9, *p* = 0.462). The model also showed that the food type factor was significant (two-way ANOVA: F_3,42_ = 5.6; *p* < 0.01). The *post hoc* Tukey multiple comparison of means test showed that *Moina* significantly grazed more *Monoraphidium* on the diets containing the strain PLANK-09. This might indicate that other cyanobacterial strains promoted higher impairment in the ability of *Moina* to feed on green algae (Figure 3, Table 2).

Regarding the sum of both food types (CR_Total_), the interaction effect between food type and the proportion of cyanobacteria in the diet on clearance rates was not significant (two-way ANOVA: F_6,36_ = 1.3, *p* = 0.277). Once again, only food type was a significant factor in these results (two-way ANOVA: F_3,42_ = 7.3; *p* < 0.001). *Post hoc* contrasts tests were similar to the observed in CRBlue post comparison tests, showing significant differences between *Microcystis* and *Planktothrix*, but not between the strains of each of these species (Figure 3, Table 2).

### 2.3. Selectivity

**Figure 4:**
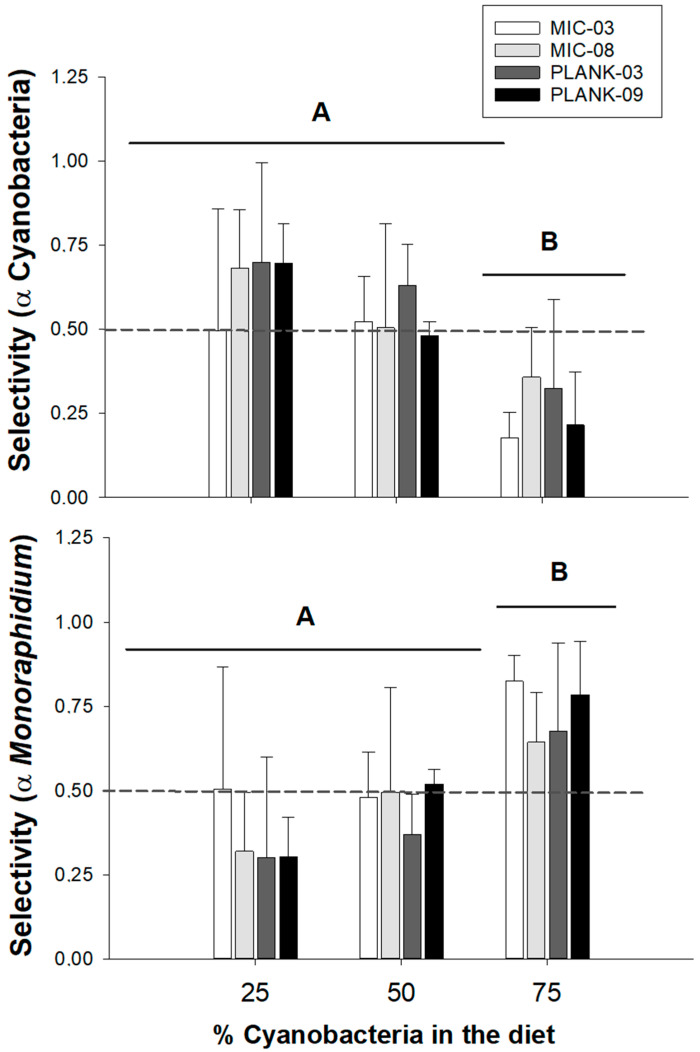
Mean selectivity coefficients of *Moina micrura* for Cyanobacteria (α Cyano) and *Monoraphidium* (α *Monoraphidium*) in mixed diets, where cyanobacteria is 75%, 50% and 25% of the total food provided (0.5 mg C L^−1^). The mixed diets were composed of cyanobacteria (*Microcystis aeruginosa* strains MIC-03 and MIC-08 and *Planktothrix isothrix* strains PLANK-03 and PLANK-09) in combination with good food, *Monoraphidium*. Error bars indicate standard deviation (n = 4). α > 0.5 (dashed line) indicates positive selection. Different letters mean significant differences, according to the tests described in Table 3.

Positive selectivity (selectivity coefficient > 0.5) of *Monoraphidium* (α *Monoraphidium*) occurred mainly in the diet with the lowest proportion of cyanobacteria in the diet (25%). Negative selectivity (or avoidance) of cyanobacteria (α Cyanobacteria) occurred in the 25 and 75% diets (Figure 4). Only the proportion of cyanobacteria in the diet had significant effects on the selectivity indexes α Cyanobacteria (two-way ANOVA: F_2,45_ = 15.1; *p* < 0.001) and α *Monoraphidium* (F_2,45_ = 13.8; *p* < 0.001). Considering the different dominances, the selection for cyanobacteria (α Cyanobacteria) was significantly lower in the 75% treatment in comparison to the 25 and 50% (Figure 4, Table 3). As for the selection for *Monoraphidium* (α *Monoraphidium*), it was higher in the highest shares of cyanobacteria on the diet when compared to 25 and 50%.

## 3. Discussion

In this study, we tested whether tropical strains of *Microcystis aeruginosa* and *Planktothrix isothrix* differ inter- and intraspecifically in losses due to grazing by the tropical cladoceran *Moina micrura*, with a history of co-occurrence of these cyanobacteria. According to our hypotheses, the type of algae and their concentrations had an interactive effect on the grazing rate of *Moina micrura*. As expected, cladocerans ingested cyanobacteria at reduced rates compared to *Monoraphidium* in single diets. The increase in concentration reduced overall grazing, except for *Monoraphidium* and the *Microcystis* strain MIC-03. In mixed diets, only food type (cyanobacterial strains) significantly affected grazing responses. Among all the tested mixed diets, the grazing responses differ inter- but not intraspecifically only on cyanobacteria and on *Monoraphidium* + cyanobacteria (total). As for the grazing on *Monoraphidium* mixed with cyanobacteria, the chlorophycean was more grazed in the presence of *Planktothrix* strain PLANK-09 in comparison to all the other diets, indicating less feeding inhibition of this strain than the others. As for feeding selectivity (α), the selection for *Monoraphidium* was significantly higher in the highest dominance of cyanobacteria (75%). Nevertheless, the type of food did not influence this index.

The incipient food level for zooplankton refers to the lowest concentration of food particles in the water at which a zooplankton individual can achieve its maximum or near-maximum feeding rate [52]. This indicates at what food concentrations zooplankton populations will begin to experience food saturation or limitation, which can impact their growth, reproduction, and survival. This threshold can vary significantly depending on the nutritional quality and palatability of the food, with profound implications for aquatic food webs [52,53]. Our results demonstrate that the incipient limiting level was determined by the quality of the food provided. In general, at all concentrations provided in single diets, we see differences between the control diet (*Monoraphidium*) and cyanobacteria. We observed the greatest feeding of *Moina* on *Monoraphidium* at a concentration of 1 mg C L^−1^, while all cyanobacteria had a negative relationship between biomass concentration and CRs, except *Microcystis* MIC 03, with a higher feeding also at the concentration of 1 mg C L^−1^. In terms of clearance rates, the onset of food saturation of *Moina micrura* at concentrations 10^9^ µm^3^ L^−1^ [38] was already reported. Our tested concentrations of carbon converted to biovolume, at the comparison level, ranged from 8 × 10^5^ µm^3^ L^−1^ to 1 × 10^7^ µm^3^ L^−1^ for all food suspensions tested in single diets. Therefore, our concentrations are well below the saturation level reported for the species; however, such concentrations were chosen based on observations of annual averages of the natural environment from which these organisms were isolated, the Jacarepaguá Lagoon [54].

Our results showed that *Moina micrura* reduced feeding on all cyanobacteria diets. This is in accordance with previous studies showing that bloom-forming cyanobacteria is a feeding deterrent for cladocerans [39,55,56,57,58]. This appears to be more acute with microcystin producer strains and larger cladocerans, such as *Daphnia* [21,59]. Microcystins are toxic cyclic peptides produced by some cyanobacteria and commonly found in freshwater and brackish environments [3,4]. In mammals, these toxins primarily affect the liver, entering cells via organic anion transporting polypeptides (OATP) [7]. Inside the cells, they inhibit protein phosphatases, leading to cytoskeletal disruption, cell death, and liver damage [7]. In cladocerans, microcystins uptake takes place in mid-gut and it is likely that, once the epithelium has been damaged, other cyanometabolites may also be absorbed and thereby result in a lethal effect [60]. Therefore, these compounds are significant stressors for human health and the integrity of water resources [3].

Microcystins appear to affect diverse aspects in zooplankton, such as their feeding, reproduction, survival and antioxidant defenses [9,32,61,62]. However, some zooplankton, especially the ones with a story of coexistence with toxic blooms, can be more tolerant to these events and even graze on *Microcystis* [15,63]. A previous study demonstrated that zooplankton from Jacarepaguá Lagoon, including high densities of *Moina micrura*, effectively accumulated microcystins from the seston, indicating some level of grazing of these cladocerans on toxic *Microcystis* [50]. The bioaccumulation of microcystin by zooplankton makes these communities an important vector for the transfer of these cyanotoxins to higher trophic levels within the aquatic food web, such as fish used for human consumption [61,64]. Cyanobacterial extracts from two Colombian reservoirs with microcystins concentrations ranging from 89 to 538 mg/g of dry biomass exhibited greater acute toxicity toward *Daphnia* clones compared to *Moina micrura* ones [59]. On the other hand, experimental assays demonstrated a significantly decreased survival of *Moina micrura* in co-occurrence with persistent cyanobacterial blooms of the Brazilian Funil Reservoir in single *Microcystis* diets [32]. The mean microcystins concentrations on the diet suspensions ranged from 53 to 477.4 ng/L, although a concentration-dependent response was not observed [32].

Our *Microcystis* strains varied strongly in microcystin production. MIC-08 cultures produced 3,4 times more microcystins compared to strain MIC-03. However, there was no significant difference in the CRs of cladocerans between diets of *Microcystis* strains in both single and mixed diets, except for the MIC-03 strain in the single diet at higher concentrations (>1 mg C L^−1^). However, it is also important to highlight the significant negative relationship between intracellular bioavailable microcystin and CRs, where only these two strains of *Microcystis* were included, as they were the only producers of this cyanotoxin. These findings may indicate that other factors in addition to microcystins may have acted in the interactions observed here. Although cyanotoxins were originally hypothesized as the primary deterrent factor to zooplankton, the toxic or inhibitory role of other metabolites has also been established [46,47,65,66,67]. Cyanobacteria traits such as large size, mucilaginous sheaths, taste/odor, toxicity, and nutritional inadequacy have already been demonstrated to act as predator defenses [8,19]. As we found, low feeding on all cyanobacteria treatments in comparison to edible *Monoraphidium*, regardless of the presence/quantity of microcystin, it is possible that a synergy of microcystin with other deterrent factors, such as morphology and other cyanobacterial metabolites, acted on feeding responses of *Moina micrura* [19,47].

In natural systems, cyanobacteria morphology, particularly the size and shape of colonies and filaments, plays a crucial role in determining grazing resistance [9,16,22]. Larger filaments and colonies are less manageable for most zooplankton, which can lead to reduced ingestion efficiency [14,51] and might explain low grazing in *Planktothrix isothrix* diets compared to *Monoraphidium*. As for *Microcystis aeruginosa*, the two strains tested in this study are unicellular or bicellular, as are most strains maintained under laboratory conditions [24]. Although this may occur in some natural environments [42,43], the most common occurrence of the species is its colonial occurrence [24,68]. Thus, the results observed here for *Microcystis aeruginosa* could have been even more outstanding if the strains had colonial morphology, adding an additional inhibitory factor to this interaction. The formation of colonies in response to predators appears to reinforce that morphology restrains grazing for some predators [69,70]. This also agrees with what was observed in other studies, which demonstrate feeding inhibition of cladocerans to *Microcystis* colonies [40,71,72]. This might be due to the feeding appendages that may become clogged by the ingestion of particles of inadequate size [40,71,73].

Contrary to our expectations, the filamentous species *Planktothrix isothrix* was significantly more grazed in the mixed diet experiments, compared to treatments with unicellular *Microcystis aeruginosa*. Furthermore, the presence of microcystin in both *Microcystis* strains appears to have promoted the strongest effects in terms of *Moina* rejection. These results are in line with the findings that filamentous cyanobacteria are significantly better foods than single-celled cyanobacteria for grazers, including cladocerans, copepods and rotifers [40,62,74]. Among *Planktothrix* strains, significant differences in the mean sizes of the two tested strains seemed to have affected feeding on good food in the mixed diets: the food suspensions with PLANK-09 had the smallest filaments, with median values 0.7 times lower than the ones of PLANK-03. PLANK-09 promoted less feeding inhibition on *Monoraphidium* than PLANK-03 in the mixed diets.

Notably, in the mixed diets, cyanobacteria avoidance and the selection of good food depended on the proportion of cyanobacteria on the diet; positive selectivity for *Monoraphidium* was observed in the highest proportion of cyanobacteria (75%). This result is unexpected, as in general, it is believed that cladocerans exhibit more passive suspension-feeding behavior, with lower prey selectivity [21,75]. Although considered mainly a generalist group, some cladocerans can actively choose and handle prey as selective or raptorial feeders [21,76]. Specially, some small cladocerans might be less generalist than the bigger ones, such as *Daphnia*, and avoid the harmful effects of cyanobacteria traits such as morphology and toxicity [10]. For instance, in experiments with natural phytoplankton, the tropical *Moina micrura* was able to graze efficiently on different sizes of particles, ranging from 2 to 40 µm equivalent spherical diameters (ESD) [38]. Furthermore, the cladocerans showed food selectivity apparently determined by particle size, although other factors regulating this selection have not been ruled out [36]. The positive selectivity coefficients observed for *Moina* in this study, along with other findings, demonstrate some degree of selectivity for a few species [37,38,77]. This is also in agreement with another study that showed that *Moina micrura* and *Daphnia laevis* had selective feeding behavior toward edible algal species, in mixed diets with a saxitoxin-producing strain of the filamentous cyanobacteria *Raphidiopsis raciborskii* [32].

The *Planktothrix isotrhrix* strains in this study were larger than those filamentous cyanobacteria tested in the previously cited studies. Furthermore, significantly higher clearance rates on this filamentous cyanobacterium were observed only in mixed diets with another suitable food type (*Monoraphidium*). In addition, a positive selectivity coefficient for *Planktothrix isothrix* was observed when *Monoraphidium* was present in high proportions (75%). Although the genus *Planktothrix* is a potential producer of microcystins [7], microcystins were not detected in the *Planktothrix* strains used in this study, which could be one less inhibitory factor for *Moina*. Our results indicate that some cyanobacteria can be ingested by tropical cladocerans even in the presence of a suitable food source.

Some studies show the coexistence of smaller body size cladocerans with cyanobacteria blooms, based on the idea that these grazers might be less likely to ingest large cyanobacterial colonies or filaments and/or develop tolerance to harmful food [21,78]. By avoiding large cyanobacteria and consuming smaller, more digestible phytoplankton, small-bodied cladocerans may gain a selective advantage in addition to facilitating cyanobacteria species [18]. On the other hand, some degree of tolerance of zooplankton to toxic cyanobacteria both in natural and laboratory conditions was already observed [15,28], allowing ingestion as well as neutralizing toxic effects [29,30]. This feeding-mode distinction helps to explain the different susceptibility of zooplankton to harmful cyanobacteria. This idea aligns with broader ecological concepts, emphasizing the role of functional traits in shaping species interactions and influencing community dynamics [21]. For instance, the inter- and intraspecific variations observed in our laboratory tests may indicate how the alternation of dominance of different species of bloom-forming cyanobacteria works in natural environments, as well as the toxicity of such blooms.

Our results align with observations in most aquatic ecosystems experiencing frequent blooms, where possibly tolerant zooplankton taxa, such as smaller cladocerans, dominate. In our study, we observed varied responses across treatments, ranging from feeding inhibition to different degrees of tolerance toward cyanobacteria, particularly non-microcystin-producing species. We also highlight the selectivity of small tropical cladocerans, a pattern that is not yet well-documented in the literature. These findings emphasize that studies incorporating phyto- and zooplankton species with a history of coexistence can provide more meaningful insights into natural ecosystem dynamics.

## 4. Materials and Methods

### 4.1. Organisms

The experiments were performed with two *M. aeruginosa* (hereafter *Microcystis*) strains (MIC-03 and MIC-08) and two *P. isothrix* (hereafter *Planktothrix*) strains (PLANK-03 and PLANK-09), isolated from a shallow tropical eutrophic coastal lagoon (Jacarepaguá Lagoon; 22° 55′ S and 43° 17′ W, Brazil) between 2009 and 2011 [79] and maintained in the culture collection of the Laboratory of Ecology and Physiology of Phytoplankton, University of Rio de Janeiro State (UERJ, Brazil). Stock cultures of the strains were maintained in a modified WC medium [80] at 25 °C, under a light intensity of 20 μmol photons m^−2^ s^−1^, provided by daylight fluorescent lamps with a photoperiod of 12:12 h light/dark cycle. The cultures were renewed weekly with fresh medium to maintain exponential growth and shaken twice daily. Under these conditions, both *Microcystis* strains were grown as single cells, not in colonies, with a mean size of 4.5 µm. *Planktothrix* strains had length and width of 700 (±217) µm × 5 (±1) µm (PLANK-03) and 520 (±476) µm × 5 (±1) µm (PLANK-09), respectively. The Mann–Whitney rank sum test indicated significant differences in the median sizes of these strains (U = 2908, *p* ≤ 0.001). The carbon content of stock cultures and food suspensions for experiments were estimated from biovolume, using the conversion formula C = aV^b^, where a = 0.1204; b = 1.051; V = biovolume, according to [81].

The cladoceran *Moina micrura* (hereafter *Moina*) was also isolated from Jacarepaguá lagoon during a mixed bloom of *Microcystis* and *Planktothrix* in 2022 and kindly provided by the Laboratory of Ecophysiology and Toxicology of Cyanobacteria (Federal University of Rio de Janeiro). Animals were kept in 1000 mL beakers filled with modified WC medium (lacking NO_3_^−^ and PO_4_^3−^) in incubators SOLAB SL-224 (SOLAB, SP, Brazil) at 25 °C, under a light intensity of 20 μmol photons m^−2^ s^−1^, provided by daylight fluorescent lamps with a photoperiod of 12:12 h light/dark cycle. The animals were fed every two days at a total carbon biomass of 0.5 mg L^−1^ with the green microalgae *Monoraphidium capricornutum* and *Ankistrodesmus stipitatus*, cultivated in the same conditions as the cyanobacteria strains. Adult individuals of *Moina* achieve a maximum length of 1.0 ± 0.1 mm.

### 4.2. Toxin Analyses of the Cyanobacterial Strains

For the microcystins (MCs) extraction procedure, 100 mL aliquots of the cyanobacterial cultures used for zooplankton grazing assays were collected. The samples were centrifuged (7200× *g*, 10 min, 4 °C) to separate the cell pellet and supernatant, representing the intracellular and extracellular fractions of the culture, respectively. The pellet was frozen at −20 °C, lyophilized, and subjected to MC extraction using a 75% methanol solution as recommended by [82]. After incubation for 1 h, the extract was centrifuged, and the supernatant obtained was dried to 75% of its initial volume. Then, solid-phase extraction (SPE) purification was performed using C_18_ bond elut cartridges (Agilent, Santa Clara, CA, USA), with the recovery of toxins in 100% HPLC-grade methanol. The samples were stored in 1.5 mL vials and subjected to MC analysis in a high-performance liquid chromatography system coupled to a photodiode array UV detector (HPLC–PDA Shimadzu, Kyoto, Japan).

The mobile phase used in the HPLC–PDA was composed of a 20 mM ammonium acetate + acetonitrile buffer solution (72:28 *v*/*v*, pH 5.0), as described in [83]. The separation was performed on a C_18_ column (5 μm—Phenomenex^®^, Torrance, CA, USA), with detection at 238 nm. The chromatographic peaks obtained were analyzed, and the UV absorption spectra were compared with the certified reference material (CRM) [D-Leu^−1^] microcystin-LR solution provided from the Cyanosol Research Group (Robert Gordon University, Aberdeen, UK). The method had the following limits of detection and quantification: LoD_concentration_ = 0.09 µg mL^−1^ and LoD_mass_ = 10 ng; LoQ_concentration_ = 0.26 µg mL^−1^ and LoQ_mass_ = 20 ng.

From HPLC analyses it was not possible to identify the presence of microcystins produced by *Planktothrix* strains, while for the *Microcystis* strains three microcystins peaks were detected based on the UV absorption spectra at 238 nm in comparison to CRM. However, these different MCs variants were not characterized for the present study. Thus, toxicity potential will be presented as the total microcystin cell quota, expressed in fg of microcystin equivalent ([D-Leu^−1^]MC-LReq) per cell and biovolume (µm^3^). The following *Microcystis* strains were characterized as producing microcystins: MIC-03 (47.57 fg[D-Leu^−1^]MC-LReq cell^−1^ or 0.36 fg[D-Leu^−1^]MC-LReq µm^3^) and MIC-08 (164.79 fg[D-Leu^−1^]MC-LReq cell^−1^ or 1.26 fg[D-Leu^−1^]MC-LReq µm^3^).

### 4.3. Experiments with Different Amounts of Food (Single Diets)

Grazing assays were performed to test the effect of different concentrations of the following food type on *Moina* clearance rates:

- *Monoraphidium capricornutum* as a good food control [55,58];
- two strains of *Microcystis aeruginosa* (MIC-03 and MIC-08) and;
- two strains of *Planktothrix isothrix* (PLANK-03 and PLANK-09).

For each algal suspension, the following concentrations were used: 0.125, 0.250, 0.5, 1, and 2 mg C L^−1^. The tests were carried out by incubating three adult animals (about 5 to 7 days old) in 24-well culture plates with 2.5 mL food suspensions, for 3 h. Controls were performed without the addition of animals. The experiments were performed in the dark, at 24 °C (±1), with four replicates per treatment.

Clearance rates of *Moina micrura* (CR, in mL ind^−1^ h^−1^) were calculated by the difference in chlorophyll-a concentrations, measured by fluorometer PHYTO-PAM (Walz, Germany), according to the method described in [84]. This method provides rapid and reliable assessments of grazing responses. It has been successfully applied across a diverse range of phytoplankton and zooplankton species [9,14,55,58,62,63], demonstrating its broad applicability and effectiveness in various ecological contexts. CRs were calculated through the following formula:

CR = {ln(Chlorophyll-a_control_ − Chlorophyll-a_treatment_)}/∆t × V/N

in which Chlorophyll-a_control_ is the final algal concentration in the controls, Chlorophyll-a_treatment_ is the final algal concentration in the treatments, ∆t—is the incubation time (h), V—is the culture volume (mL) and N—is the number of animals.

In all grazing experiments, cladocerans were added to the food suspensions after 12 h of starvation, to minimize potential variations in grazing responses attributed to differences in gut fullness. All animals used were alive and swimming actively at the end of the experiments and were removed prior to chlorophyll quantification in PHYTO-PAM.

### 4.4. Experiments with Mixed Diets

To assess the effects of cyanobacterial relative dominance, the second grazing experiment involved feeding *Moina* with mixtures of cyanobacteria strains (*Microcystis* and *Planktothrix*) and *Monoraphidium*. Cyanobacteria in the zooplankton diet was provided as 25%, 50%, and 75% of total food (by carbon) relative to a *Monoraphidium* proportion of 75%, 50% and 25% in a total food concentration of 0.5 mg C L^−^^1^. In this design, we aim to comprehend the effects of cyanobacteria dominance (% on the mixed diets with *Monoraphidium*) on the clearance rate of (A) cyanobacteria (CRBlue), (B) *Monoraphidium* (CRGreen) and (C) sum of both food types (CRTotal). Each treatment in the mixed food experiments also had four replicates and was performed as described for single diets.

Grazing selectivity is characterized by the difference between the relative consumption of a food type and its availability in the food suspensions. Positive selection is observed when a specific food item is ingested at a higher proportion than its relative abundance. For these experiments, we calculated the selectivity coefficient (α) estimated from the Ivlev’s ratio for each food type, based on chlorophyll-α variations, as described in [85], according to the following formula:

I_i_ = r_i_ × n_i_^−1^

where r_i_ is ratio of the CR on food type i to the sum of the CR on both foods and n_i_ is proportion of i in the food mixture. However, the result of this formula is not normalized. Therefore, we quantified food selection using selectivity coefficient, α. This coefficient effectively standardizes Ivlev’s ratio, providing a clear measure of selectivity ranging from 0 to 1. Selectivity (α) was then calculated for each food type (blue or green) as follows:

α_Blue_ = I_Blue_ (I_Blue_ + I_Green_)^−1^

α_Green_ = I_Green_ (I_Green_ + I_Blue_)^−1^

The selectivity for Blue (Cyanobacteria) was calculated for each strain tested in this study (MIC-03, MIC-08, PLANK-03 and PLANK-09). This method has proven effective for comparing zooplankton grazing selectivity in similar experimental designs [86]. For a given food type, α can range between zero and one, with values > 0.5 indicating positive selection, α = 0.5 indicating no selection, and α < 0.5 indicating prey avoidance.

### 4.5. Statistical Analysis

For the single diet experiments, we tested the effect of food types and tfood concentration on the clearance rates (by grazing rate of *Moina micrura*). All data was transformed (log_10_). We used generalized linear models (GLM) with a gaussian family function. We used the package “fitdistrplus” [87] to assess the empirical distribution of the data. For the mixed diet experiments, we used three two-way ANOVA to test the effect of food type and of the proportion of cyanobacteria in the diet on clearance rates on cyanobacteria (CRBlue), on *Monoraphidium* (CRGreen), and on the sum of both food types (CRTotal). The CRGreen and CRTotal were log-transformed to improve the normality of the data and their residual distribution. Furthermore, we used a two-way ANOVA to test the effect of food type and of the proportion of cyanobacteria on the selectivity of *Moina micrura* in grazing *Monoraphidium*. Post-hoc Tukey’s Honest Significant Difference (Tukey HSD) was used to test significant differences in the case of significant result in the two-way ANOVA with the package “multcomp” [88]. All statistical analyses were performed using the R program [89]. Linear regression analysis was performed to assess the relationship between filtration rates and bioavailable microcystin in the single diets. Bioavailable microcystin was calculated as the product of the cellular quota and cell density for each carbon concentration. This analysis was performed with the averages of the CRs and data only from the microcystin-producing strains, MIC-03 and MIC-08. The regression analysis was conducted using SigmaPlot software v 14.0.

## Figures and Tables

**Table 1:** Generalized linear model (GLM) results showing the effect of food type and food concentration (logarithmized data) on the clearance rates (also logarithmized data) in the functional response experiments. The Chlorophycean *Monoraphidium* was the reference group.

Coefficients	Estimate	SE	t Value	*p*
Intercept	−0.60	0.09	−6.66	<0.001
Log 10 (Concentration)	−0.19	0.17	−0.11	0.91
*Monoraphidium* vs. MIC-03	−0.62	0.13	−4.86	<0.001
*Monoraphidium* vs. MIC-08	−1.02	0.13	−8.04	<0.001
*Monoraphidium* vs. PLANK-03	−1.03	0.13	−8.09	<0.001
*Monoraphidium* vs. PLANK-09	−1.09	0.13	−8.52	<0.001
Concentration: *Monoraphidium* vs. MIC-03	−0.02	0.24	−0.07	0.94
Concentration: *Monoraphidium* vs. MIC-08	−0.78	0.24	−3.19	<0.001
Concentration: *Monoraphidium* vs. PLANK-03	−0.52	0.24	−2.12	<0.01
Concentration: *Monoraphidium* vs. PLANK-09	−0.65	0.24	−2.67	<0.001

**Table 2:** Post-hoc Tukey’s Honest Significant Difference of clearance rates between food type in mixed diet experiment. (**A**) cyanobacteria (CRBlue), (**B**) *Monoraphidium* (CRGreen), and (**C**) sum of both food types (CRTotal).

**A.** **CRBlue**	**Estimate**	**SE**	**t Value**	***p***
MIC-03: MIC-08	0.002	0.13	0.19	0.85
MIC-03: PLANK-03	−0.03	0.13	−2.68	<0.05
MIC-03: PLANK-09	−0.03	0.13	−2.50	<0.05
MIC-08: PLANK-03	0.37	0.13	2.87	<0.01
MIC-08: PLANK-09	0.03	0.13	2.87	<0.05
PLANK-03: PLANK-09	−0.002	0.13	−0.18	0.85
**B.** **CRGreen**	**Estimate**	**SE**	**t Value**	***p***
MIC-03: MIC-08	−0.21	0.11	−1.85	0.07
MIC-03: PLANK-03	0.02	0.11	0.14	0.89
MIC-03: PLANK-09	0.25	0.11	2.25	<0.05
MIC-08: PLANK-03	0.23	0.11	1.99	0.05
MIC-08: PLANK-09	0.47	0.11	4.09	<0.001
PLANK-03: PLANK-09	0.24	0.11	2.10	<0.05
**C.** **CRTotal**	**Estimate**	**SE**	**t Value**	***p***
MIC-03: MIC-08	−0.08	0.08	−0.91	0.37
MIC-03: PLANK-03	0.18	0.08	2.18	<0.05
MIC-03: PLANK-09	0.27	0.08	3.23	<0.01
MIC-08: PLANK-03	0.26	0.08	3.09	<0.01
MIC-08: PLANK-09	0.35	0.08	4.14	<0.001
PLANK-03: PLANK-09	0.09	0.08	1.05	0.30

**Table 3:** Post-hoc Tukey’s Honest Significant Difference of the selectivity index (α) between the proportion of cyanobacteria in the diet in mixed diet experiment. (**A**) Selectivity index for cyanobacteria. (**B**) Selectivity index for *Monoraphidium*.

**A.** **α** **Cyanobacteria**	**Estimate**	**SE**	**t Value**	*p*
50:25	−0.11	0.07	−1.54	0.13
75:25	−0.37	0.07	−5.34	<0.001
75:50	−0.27	0.07	−3.79	<0.001
**B.** **α** ***Monoraphidium***	**Estimate**	**SE**	**t Value**	***p***
50:25	0.11	0.07	1.54	0.13
75:25	0.37	0.07	5.33	<0.001
75:50	0.27	0.07	3.79	<0.001

## Data Availability

The original contributions presented in this study are included in this article. Further inquiries can be directed to the corresponding authors.
